# Ocular pharmacokinetics of intravitreal conbercept in a rabbit model following retinal scatter laser photocoagulation

**DOI:** 10.3389/fphar.2025.1534048

**Published:** 2025-04-07

**Authors:** Lanlan Chen, Hua Liu, Qingrong Zhang, Houbin Huang

**Affiliations:** ^1^ Medical School of Chinese PLA, Beijing, China; ^2^ Senior Department of Ophthalmology, The Third Medical Center of Chinese PLA General Hospital, Beijing, China

**Keywords:** ocular pharmacokinetics, photocoagulation, conbercept, anti-VEGF, rabbit

## Abstract

**Objective:**

The present study aims to evaluate the ocular pharmacokinetics of intravitreal conbercept after retinal scatter laser photocoagulation.

**Methods:**

Thirty male Chinchilla rabbits (60 eyes) were used in this study. The control and photocoagulated animals received single bilateral intravitreal injections of conbercept, and the ocular tissues were collected and quantified for drug concentration using ELISA. Statistical analysis was then performed to compare the pharmacokinetic parameters between the control and photocoagulated eyes.

**Results:**

The conbercept concentrations were higher in the control rabbits than the photocoagulated rabbits and reached peak values in all ocular tissues 1 d after intravitreal dosing. The terminal t_1/2_ values in the vitreous humor (4.36 d), aqueous humor (4.19 d), retina (3.94 d), and choroid-RPE (3.84 d) of the control eyes were longer than those in the photocoagulated eyes (3.82 d, 3.69 d, 3.65 d, and 3.58 d, respectively). Conbercept exposure assessed using AUC_0-t_ was lower in the photocoagulated rabbits than control animals in all four ocular matrices (*p* < 0.01). The clearance and volume of distribution were greater in the photocoagulated eyes than the control eyes, while the mean residence times were shorter in all four matrices.

**Conclusion:**

Retinal scatter laser photocoagulation shortly before single intravitreal injection of conbercept enabled higher drug clearance and shorter half-life values, resulting in lower exposure in the ocular tissues compared to non-photocoagulated conditions. The distinct ocular pharmacokinetics of intravitreal conbercept observed in a rabbit model through retinal scatter laser photocoagulation is expected to enlighten further studies on investigating the optimal order of the combination of photocoagulation and anti-VEGF agents.

## 1 Introduction

Anti-vascular endothelial growth factor (anti-VEGF) agents have revolutionized the management of retinal vascular disorders characterized by pathological neovascularization or abnormal vascular permeability (Andreoli and Miller, 2007); hence, they have emerged as the first-line therapies for conditions such as diabetic macular edema (DME) and macular edema (ME) secondary to retinal vein occlusion (RVO). Conbercept is a fully humanized soluble VEGF-binding Fc fusion protein that exhibits high affinities for VEGF-A, VEGF-B, and placental growth factor; these are attributed to its unique structures of the extracellular domain 2 of vascular endothelial growth factor receptor 1 (VEGFR1) and extracellular domains 3 and 4 of VEGFR2, which enable more efficient reduction of vitreous VEGF levels and inhibition of neovascularization (Chen et al., 2013; Sun et al., 2017; Liu et al., 2022; Liu et al., 2019).

As outlined in the latest guidelines on clinical practices, panretinal photocoagulation (PRP) may be more suitable as an initial therapeutic intervention for patients with proliferative diabetic retinopathy (PDR), particularly under poor compliance conditions (Jampol et al., 2020), and remains the primary treatment in ischemic central retinal vein occlusion complicated by new vessels in the iris or angle (Scott et al., 2020; Nicholson et al., 2022). A combination of PRP and anti-VEGF agent could be a more optimal strategy in such conditions (Nicholson et al., 2022; Figueira et al., 2018), and more than one-third of ophthalmologists favor a combined strategy in clinical practice (i.e., real-world settings) (Singh, 2018; Singh and Hahn, 2019). However, there is no consensus regarding the optimal order in combined treatment. Although anti-VEGF administration prior to PPR could favor relief from ME, prompt recovery of visual acuity, and avoidance of ME severity induced by PRP, some practitioners choose to commence PRP prior to anti-VEGF for exuberant iris or angle new vessels in RVO or any imminent vitreous hemorrhage in severe PDR (Filho et al., 2011; Messias et al., 2012). Some clinicians opt to administer anti-VEGF agents at the end of vitrectomy combined with endolaser photocoagulation for PDR to reduce postoperative vitreous hemorrhage (Ahn et al., 2011; Sun et al., 2023). Thus far, only three retrospective studies have explored the order of anti-VEGF therapy and PRP in the treatment of DME secondary to severe non-proliferative diabetic retinopathy (NPDR) or PDR (Kartasasmita and Harley, 2019; Zhang et al., 2021; Alsoudi et al., 2024); however, these have yielded somewhat divergent results, and the underlying mechanisms remain to be elucidated (Zhang et al., 2021).

After intravitreal administration, drug elimination occurs via blood–ocular barriers and aqueous humor outflow to systemic circulation. High amounts of VEGF-binding Fc fusion protein were detected in the retinal and choroidal tissues after intravitreal injection into healthy rabbit eyes, indicating involvement of these tissues in the clearance route of VEGF-binding reagents (Joo et al., 2017). The endothelia of the retinal capillaries (inner blood–retinal barrier or inner BRB) and retinal pigment epithelium (RPE; outer blood–retinal barrier or outer BRB) are major barriers to macromolecule elimination from the vitreous space (Pitkänen et al., 2005; Del Amo et al., 2017). Retinal laser photocoagulation might open new pathways in the outer BRB for fluid and molecule transportation between the retina and choriocapillaris (Wallow, 1984; Peyman et al., 1971a, 1971b; Peyman and Bok, 1972). Moreover, scatter photocoagulation could increase leukocyte rolling in the retina, which is known to be involved in the breakdown of the inner BRB, resulting in retinal thickening and ME (Nonaka et al., 2002; Er et al., 2000; McDonald and Schatz, 1985; Tsujikawa et al., 1999). Deissler et al. (2020) observed that increased amounts of aflibercept pass through a monolayer of VEGF-A-challenged immortalized bovine retinal endothelial cells, likely owing to enhanced paracellular flow. It is not yet clear whether the drug clearance of VEGF-binding Fc fusion protein from the vitreous space is affected under impaired BRB conditions after scatter laser photocoagulation. In the present study, we assessed the distribution of intravitreally administered conbercept in the ocular tissues of non-photocoagulated and photocoagulated rabbit eyes to further analyze the impacts of photocoagulation on conbercept from the perspective of pharmacokinetics.

## 2 Materials and methods

### 2.1 Antibodies and reagents

The engineered protein conbercept (KH902; Chengdu Kanghong Biotech Co., Sichuan, China) was purchased, and aliquots of the antibody solution were stored in inert plastic vials for less than 4 weeks.

### 2.2 Animals

The animal experiments were conducted in conformity to the guidelines of the Association for Research in Vision and Ophthalmology Statement for the Use of Animals in Ophthalmic and Vision Research. Thirty male Chinchilla rabbits (Qingdao Kangda Aibo Biotechnology Co., Ltd., Shandong, China) weighing 2.0–2.4 kg were housed in stainless-steel cages with free access to food and water. The animal room was maintained at a temperature of 20°C–25°C, with a relative humidity of 40%–70% and 12-h light/dark cycles. All animals were acclimated to the surroundings for at least 1 week before commencing the experiments.

Prior to the procedures, a slit-lamp microscopy and an ophthalmoscopy were performed on the animals to confirm that all eyes were normal. The rabbits were then divided randomly into the control (n = 15 receiving single bilateral intravitreal injections of conbercept) and photocoagulation (n = 15 receiving bilateral retinal scatter laser photocoagulation 3 d before single intravitreal injection of conbercept) groups. Three rabbits (six eyes) were used at each time point (1, 4, 7, 14, and 28 d) from each group and were euthanized following intravitreal injection.

### 2.3 Retinal laser photocoagulation

Transpupillary retinal laser photocoagulation was performed on both eyes of the rabbits. Pupillary dilatation was achieved using 0.5% tropicamide and 2.5% phenylephrine hydrochloride. The rabbits were anesthetized using isoflurane and 0.4% oxybuprocaine hydrochloride ophthalmic drops topically on the eyes. A standard retinal laser contact lens was used to focus the laser light on the rabbit fundus. Grade III lesions were expected as the spot consisted of a grayish-white retinal discoloration with distinct central whitening, according to the Tso grading system. The laser power setting was 50–90 mW adjusted to the laser spot response, and the duration of exposure was 150 ms to yellow wavelength (577 nm); the spacing between the coagulation spots were adjusted to the width of approximately one spot. The spots were placed at a distance of one-disk diameter peripherally from the optic disk and medullary rays, and the number of laser spots per eye was maintained constant at 500. Photocoagulation was carefully carried out by an experienced ophthalmologist (CLL).

### 2.4 Intravitreal injection

Before the intravitreal injection, the rabbits were administered tobramycin drops in the fornices of the eyes at least six times. After the animal was generally and topically anesthetized as above, a self-retaining eyelid speculum was used to stabilize the eyelid and 5% povidone iodine was placed on the conjunctival sac. Then, a 31-gauge needle washed with saline solution was inserted intravitreally approximately 1.5 mm (measured by ophthalmic calipers) behind the superior nasal limbus, and the needle tip was directed to the mid-vitreous space to a depth of approximately 6 mm. A total of 0.5 mg (0.05 mL) of conbercept was then administered slowly, and the needle was maintained steadily for 20 s before withdrawal to reduce drug leakage. After withdrawal, we immediately compressed the injection site with a sterile cotton-tip applicator for 30 s to promote wound healing. The entire procedure was conducted carefully by an experienced ophthalmologist (CLL). Ofloxacin eye ointment was then applied twice topically immediately after the intravitreal injection. The treated eyes were monitored daily for signs of inflammation.

### 2.5 Sample collection

To study the effects of retinal laser photocoagulation on intraocular conbercept after intravitreal injection, we measured the amounts present in the aqueous humor, vitreous humor, retina, and choroid-RPE in the control and photocoagulated groups on days 1, 4, 7, 14, and 28 after injection. Both eyes of three rabbits at each time point were subjected to the same procedure of photocoagulation and intravitreal injection; thus, the number of eyes in each group at each time point was six. The rabbits were euthanized via intravenous injection of 100 mg/kg of sodium pentobarbital. The aqueous humor was withdrawn from the anterior chamber using a 29-gauge single-use insulin syringe with the needle bent to avoid the iris and lens; the sample was then centrifuged immediately at 3000 × *g* for 10 min at 4°C to measure the volume. The eye was then enucleated through a conjunctival incision by cutting the extraocular muscles close to their insertion and the optic nerve. After washing the eyeball with ice-cold phosphate-buffered saline (PBS) buffer, we quickly removed the remaining soft tissue to facilitate access to the sclera. Then, a 360° cut was made 2 mm posterior to the limbus to separate the anterior part of the eye from the posterior, while the vitreous space remained adhered to the anterior part and completely detached from retina. The vitreous samples were mixed with a protease inhibitor cocktail (P8340, Sigma-Aldrich, St. Louis, MO, United States) at a ratio of 40 mg to 1 μL before being chopped and homogenized on ice. The samples were then centrifuged at 14000 × *g* at 4°C for 10 min, and the supernatant volume was measured. After cutting the posterior eye cup into four parts, we gently separated the retinal tissues into two layers, namely, the neural retina (NR) and retinal pigment epithelium/Bruch membrane/choriocapillaris (RPE-choroid). Both tissue types were weighed, chopped, and homogenized using a protein extraction kit (P0013M, Beyotime Biotechnology, Shanghai, China) containing a protease inhibitor cocktail before being centrifuged at 14000 × *g* at 4°C for 10 min. All the dissection procedures were conducted within 30 min under a stereomicroscope and on ice to minimize tissue damage, and all samples were frozen at −80°C until testing.

### 2.6 Measurement of conbercept concentrations in the ocular tissues

The protein concentrations were measured with the BCA kit (P0012S, Beyotime Biotechnology, Shanghai, China), and the conbercept concentration in each sample was measured using an indirect enzyme-linked immunosorbent assay (ELISA). The recombinant human vascular endothelial growth factor-A (VEGF165, #100-20, PeproTech) was used for capturing, and the goat anti-human IgG/Fc secondary antibody labeled with horseradish peroxidase (ab97225; Abcam, Cambridge, UK) was used for detection. Briefly, the human VEGF_165_ protein was diluted to a concentration of 0.125 μg/mL in PBS and immobilized in 96-well microplates (100 µL/well, Corning Inc., Corning, NY, United States). After overnight incubation at 4°C, the microplates were washed three times using PBS with Tween 20 (PBST) and blocked using 1% bovine serum albumin (BSA) in PBS for 1 h at 37°C ± 1°C. After the final washing, the plates were dried and stored at 4°C. Then, the aqueous humor, vitreous humor, retina, RPE-choroid, and serum samples were diluted in PBST and aliquoted into the rVEGF_165_-coated microplates (100 µL/well) on the same day; the microplates were then incubated for 2 h at 37°C ± 1°C with agitation and washed three times with PBST. For each plate, a standard curve was constructed using known conbercept concentrations (0, 25, 50, 100, 200, 400, 800, and 1,600 ng/mL) to estimate the conbercept concentration in each sample. The diluted secondary antibody (50 ng/mL) was aliquoted onto the microplates, and the plates were shaken for 1 h at 37°C ± 1°C, followed by washing. After treating the tetramethylbenzidine substrate with hydrogen peroxide in the dark for 10–20 min, the reaction was stopped using 2 mol/L of phosphoric acid. The optical density was determined by detecting the absorbance at 450 nm with the reference at 570 nm. The conbercept concentrations in our samples were calculated from the four-parameter logistic curve fit of the same standard curve. The final concentrations in the aqueous humor and vitreous humor were expressed in terms of nanogram per milliliter (ng/mL), and the concentrations in the retina and RPE-choroid samples were calculated by dividing the weight of conbercept (µg) by those of the corresponding tissues (g).

## 3 Pharmacokinetic analysis

The pharmacokinetic parameters in different matrices were calculated using the sparse sampling computations of non-compartmental analysis available in Phoenix WinNonlin (version 8.1; Pharsight Corporation, Mountain View, CA, United States).

## 4 Statistical analysis

An independent-sample t-test was performed using SAS (Version 9.4; SAS Institute, Inc., Cary, NC) to compare the exposure pharmacokinetic parameters, including peak plasma concentration (C_max_) and area under the concentration–time curve from time 0 to the last sampling time (AUC_0-t_), for conbercept in the control versus photocoagulated groups. A two-way ANOVA was performed using GraphPad Prism (Version 10.1.2) to compare the decreases in conbercept concentrations with time in the different matrices. Given the sampling limitations and poor linear fit in the elimination phase of the concentration–time curve, statistical comparisons of the elimination half-life (t_1/2_), clearance (CL), volume of distribution (V), and mean residence time (MRT) between the two groups could not be performed.

## 5 Results

### 5.1 General health and clinical findings

The animals gained weight continuously during the study and showed no differences between the groups. No clinical signs or changes were observed in food consumption or bodyweight attributable to the drug. Transient conjunctival injection and mild discharge were observed (12/30 eyes) after retinal scatter photocoagulation but diminished within a day. Retinal edema was noted in the eyes of the photocoagulated group 1 d after intravitreal administration, as determined by necropsy. No ocular inflammation was observed in the control group.

### 5.2 Ocular pharmacokinetics of conbercept after intravitreal administration in the control and photocoagulated eyes

**FIGURE 1:**
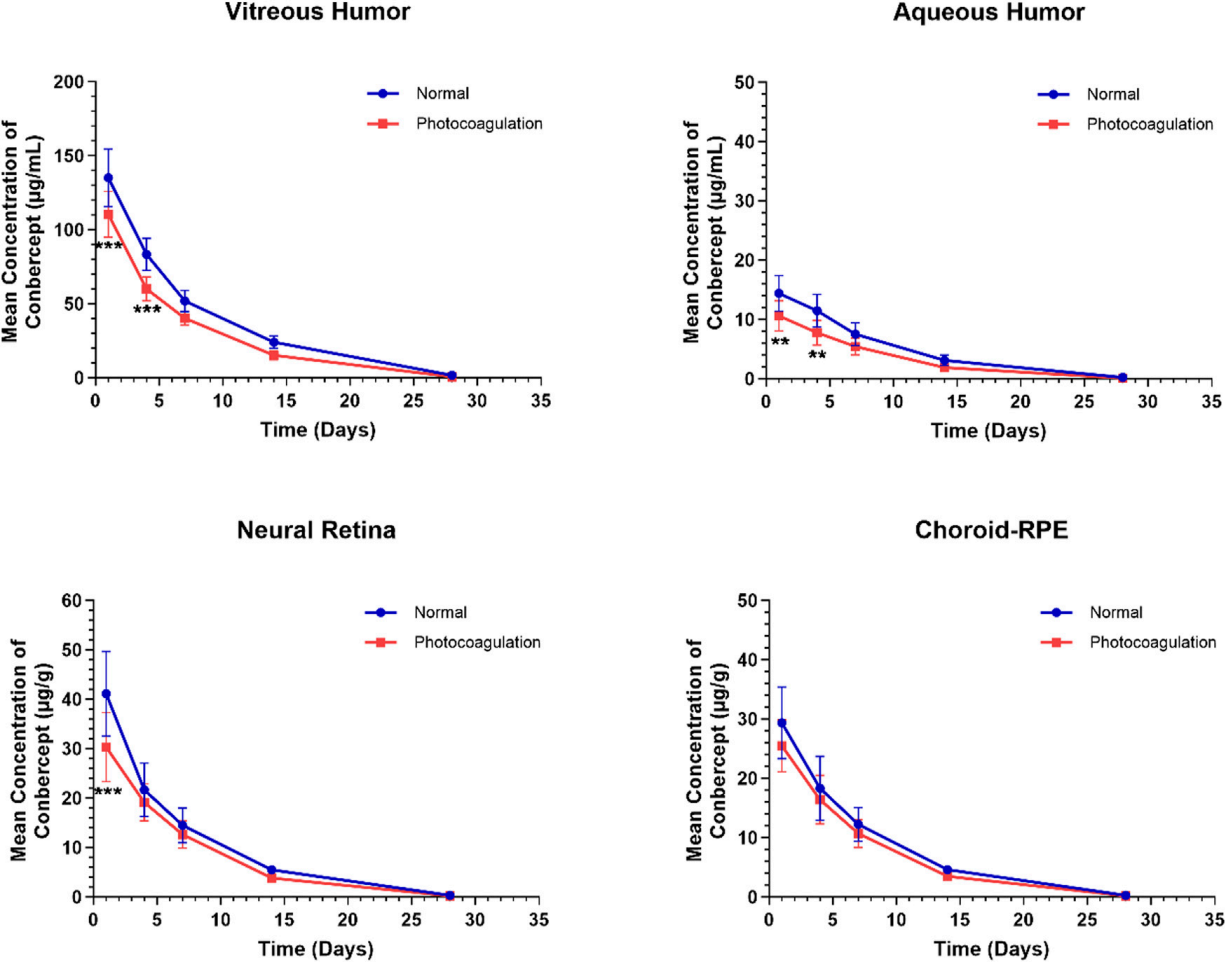
Mean (± standard deviation) concentrations of conbercept in the vitreous humor, aqueous humor, neural retina, and choroid-RPE in normal and photocoagulated rabbits following a single intravitreal injection of 0.5 mg of conbercept. n = 6 eyes/time point.

Figure 1 shows the concentration–time profiles of conbercept in the ocular tissues following a single intravitreal injection of 0.5 mg in the bilateral eyes of the control and photocoagulated rabbits. Generally, the conbercept concentrations were higher in the control rabbits than photocoagulated rabbits, and the concentrations reached peak values in all ocular tissues 1 d after intravitreal dosing, before decreasing monoexponentially. In the photocoagulated group, the C_max_ values were 110.34 ± 15.62, 10.60 ± 2.55, and 30.31 ± 6.99 μg/g in the vitreous humor, aqueous humor, and retina, respectively, which were significantly lower than those in the control group (135.10 ± 19.39, 14.41 ± 3.02, and 41.15 ± 8.56 μg/g, respectively). Significant concentration differences were observed in the vitreous humor and aqueous humor until 4 d after intravitreal injection in the two groups. No significant concentration differences were observed in the choroid-RPE in the control and photocoagulated rabbits.

The ocular pharmacokinetic parameters for conbercept in the control and photocoagulated rabbits are listed in Table 1. Conbercept exposures assessed using the AUC_0-t_ were lower in all four ocular matrices in the photocoagulated than control animals: vitreous humor (*p* < 0.001), aqueous humor (*p* < 0.001), retina (*p* < 0.01), and choroid-RPE (*p* < 0.01). The observed magnitude of difference in ocular exposure (ratio between control and photocoagulated) was less in the choroidal-RPE than in the other three matrices. Conbercept concentrations in the ocular tissues decreased in accordance with a first-order kinetic model, with terminal t_1/2_ values of 4.36 d, 4.19 d, 3.94 d, and 3.84 d in the vitreous humor, aqueous humor, retina, and choroid-RPE in the control eyes being longer than those (3.82 d, 3.69 d, 3.65 d, and 3.58 d) in the photocoagulated eyes, respectively. In the photocoagulated eyes, the CL and V were greater than those in the control eyes, while the MRT was shorter for all four matrices.

**TABLE 1:** Pharmacokinetic parameters for conbercept in rabbit eyes following a single intravitreal injection of 0.5 mg of conbercept.

Matrix	Pharmacokinetic parameters	Normal	Photocoagulation	Exposure ratio, normal/photocoagulation	*p* value
Vitreous humor	C_max_, µg/mL	135.10 ± 19.39	110.34 ± 15.62	1.22	<0.05
	AUC_0-tlast,_ d*µg/mL	1,045.18 ± 31.42	767.19 ± 23.09	1.36	<0.001
	t_max_, d	1	1	N/A	N/A
	t_1/2_, d	4.36	3.82	N/A	N/A
	V, mL	2.98	3.57	N/A	N/A
	CL, mL/d	0.47	0.65	N/A	N/A
	MRT_last_, d	6.67	6.18	N/A	N/A
Aqueous humor	C_max_, µg/mL	14.41 ± 3.02	10.60 ± 2.55	1.36	<0.05
	AUC_0-tlast_, d*µg/mL	134.99 ± 6.91	92.68 ± 5.24	1.46	<0.001
	t_max_, d	1	1	N/A	N/A
	t_1/2_, d	4.19	3.69	N/A	N/A
	V, mL	22.16	28.50	N/A	N/A
	CL, mL/d	3.67	5.36	N/A	N/A
	MRT_last_, d	6.88	6.56	N/A	N/A
Neural retina	C_max_, µg/g	41.15 ± 8.56	30.31 ± 6.99	1.35	<0.05
	AUC_0-tlast_, d*µg/g	279.29 ± 12.33	222.60 ± 9.59	1.25	<0.01
	t_max_, d	1	1	N/A	N/A
	t_1/2_, d	3.94	3.65	N/A	N/A
	V, g	10.12	11.76	N/A	N/A
	CL, g/d	1.78	2.23	N/A	N/A
	MRT_last_, d	6.14	6.01	N/A	N/A
Choroid-RPE	C_max_, µg/g	29.34 ± 6.07	25.49 ± 4.39	1.15	0.23
	AUC_0-tlast_, d*µg/g	224.87 ± 10.51	191.58 ± 8.14	1.17	<0.01
	t_max_, d	1	1	N/A	N/A
	t_1/2_, d	3.84	3.58	N/A	N/A
	V, g	12.25	13.42	N/A	N/A
	CL, g/d	2.21	2.60	N/A	N/A
	MRT_last_, d	6.37	6.12	N/A	N/A

C_max_, observed maximum concentration, mean ± standard deviation; t_max_, time to C_max_; CL, clearance; V, volume of distribution; MRT, mean residence time; AUC_0-tlast_, area under the curve until the last measurable concentration, mean ± standard error; n = 6 eyes/time point; N/A, not applicable.

## 6 Discussion

PRP and anti-VEGF therapy are the treatment options for patients with ischemic retinopathy, including PDR, severe NPDR, and RVO complicated by neovascularization (Scott et al., 2020; Figueira et al., 2018; Gross et al., 2015). Different guidelines recommend that anti-VEGF injection be combined with PRP in certain indications (Jampol et al., 2020; Scott et al., 2020; Nicholson et al., 2022). As shown by the ASRS PAT surveys, combined therapy for the treatment of PDR has gained popularity in clinical practice (Singh, 2018; Singh and Hahn, 2019). In particular, to the best of our knowledge, no studies have assessed the impacts of retinal scatter photocoagulation on the ocular pharmacokinetics of intravitreal Conbercept.

Li et al. (2012) and Di et al. (2021) investigated the ocular pharmacokinetics of normal rabbits following a single intravitreal injection of conbercept (0.5 mg). Their results showed that the peak concentrations in vitreous humor were 249.22 μg/mL after 0.5 d and 126.25 μg/mL after 1 d of intravitreal injection, but both studies reported a half-life of 4.24 d. In our study, we obtained a similar half-life for conbercept in the vitreous humor of the control rabbits (4.36 d), and the peak concentration of 135.10 ± 19.39 μg/mL was observed in the vitreous humor 1 d after intravitreal injection. The half-life of conbercept in the aqueous humor in our study was higher than that reported by Li et al. but lower than that noted by Di et al., which may be related to the different breed of experimental rabbits and ELISA used. The present study shows that scatter retinal photocoagulation (at 3 d) shortly before conbercept injection could lead to increased drug clearance and shorter half-life, resulting in less exposure to the vitreous humor, aqueous humor, and retina compared to the non-photocoagulation group. Substantial blood volume and rapid blood flow velocity are believed to be the reason for insignificant C_max_ values in the choroidal tissue.

Recently, Alsoudi et al. (2024) suggested that the sequence of treatment in combination therapy may impact patient outcomes in large-scale retrospective studies; they reported that patients treated with PRP prior to anti-VEGF injections had higher risks of vitreous hemorrhage, tractional retinal detachment, or progression to pars plana vitrectomy compared to those treated with anti-VEGF injections prior to PRP. In another small retrospective study, Zhang et al. (2021) noted no significant differences in the visual and anatomical outcomes in the compared groups but observed that PRP after intravitreal conbercept required fewer injections than prior PRP administration at 1 and 2 years follow-up. In addition to speculating that timely anti-VEGF administration can rapidly suppress VEGF activities in the retina and vitreous humor, reduce vascular permeability, and promote the resorption of intraretinal or subretinal fluid, we demonstrate significant differences in drug exposures following intravitreal conbercept administration in the present study along with prior scatter retinal photocoagulation treatment inducing higher drug clearance and shorter half-life of conbercept; these procedures would result in less powerful inhibition of VEGF and require more frequent drug injections as well as highlight the mechanisms underlying the distinct prognosis.

Thermal destruction by retinal photocoagulation triggers focal inflammation in the retinal tissue. Leukocyte rolling and inflammatory factors, including IL-6, VEGF, and TNFα, have been shown to increase after scatter laser photocoagulation in both the photocoagulated and untreated portions of the retina, resulting in augmented vascular permeability and retinal edema (Nonaka et al., 2002; Er et al., 2000; Masahiko et al., 2009; Itaya et al., 2007). VEGF and its combination with TNFα and IL-1β can disrupt retinal endothelial barrier integrity remarkably *in vitro*, inducing permeability to large molecules and even aflibercept (Deissler et al., 2020; van der Wijk et al., (2017)). We hypothesize that the enhanced permeability to conbercept within the inner BRB triggered by abundant inflammatory factors following scatter photocoagulation may contribute to the observed increase in drug clearance. Additionally, neonatal Fc receptor (FcRn) expression has been proven to be upregulated in laser-photocoagulated rat retina, potentially mediated by TNFα (Kim et al., 2009), while the role of FcRn in the uptake and transport of Fc-containing molecules has been firmly established in RPE and retinal endothelial cells (Dithmer et al.,2016; Deissler et al., 2017). Accordingly, by integrating these two lines of evidence, one can excuse the possibility of a relationship between FcRn upregulation and lower conbercept exposure in scatter photocoagulation. Moreover, laser photocoagulation disrupts the external limiting membrane and RPE, thereby extending the outer nuclear layer into Bruch’s membrane and the choroid Fukuchi et al., (2001). Intravitreal peroxidase has been shown to diffuse rapidly through all layers of a 3-week-old photocoagulated scar in contact with the Bruch’s membrane (Peyman et al., 1971b). This raises the question of whether conbercept can freely traverse the compromised RPE barrier in a fresh photocoagulated spot. Hence, further research is warranted to elucidate the underlying mechanisms.

Irrespective of the findings, there are some limitations to our study. First, the interval between photocoagulation and intravitreal injection in our study may not fully replicate clinical practice. Owing to ethical considerations regarding animal welfare and the principle of minimal use, a 3-d research interval was selected herein given the pronounced laser-induced damage observed during this period. Second, the pharmacokinetics observed in rabbits may not be entirely comparable to those in humans given the anatomical disparities between rabbit and human eyes. Finally, normal healthy rabbit eyes were utilized in our study, and an animal model with ischemic retinopathy, such as diabetic retinopathy or RVO was not established; this is another avenue for further exploration in the future.

## Data Availability

The original contributions presented in this study are included in the article/supplementary material, and any further inquiries may be directed to the corresponding author.
